# Efficacy Analysis of Percutaneous Endoscopic Lumbar Discectomy Combined with PEEK Rods for Giant Lumbar Disc Herniation: A Randomized Controlled Study

**DOI:** 10.1155/2020/3401605

**Published:** 2020-03-10

**Authors:** Xiang Gao, Kaiying Tang, Yu Xia, Xun Zhang, Keran Wang, Zhengjian Yan, Yu Du, Liang Chen

**Affiliations:** Department of Orthopedics, The Second Affiliated Hospital of Chongqing Medical University, No. 76 Linjiang Road, Yuzhong District, Chongqing 400010, China

## Abstract

**Objective:**

This study describes a randomized controlled trial that assesses percutaneous endoscopic lumbar discectomy (PELD) combined with a polyetheretherketone (PEEK) rod in patients with GLDH (herniation affecting 50% of the sagittal diameter of the spinal canal) and reports the 2-year follow-up outcome.

**Methods:**

In all, 243 patients were randomly assigned to undergo PELD or PELD combined with a PEEK rod by generating random numbers with a random number generator. Clinical outcome data, including the numerical rating scale (NRS), were used to assess the patients' back and leg pain, while the Oswestry Disability Index (ODI) was used to quantify pain and disability. Imaging data included intervertebral disc height (IDH), range of motion (ROM), and modified Pfirrmann grades.

**Results:**

At the final follow-up, the NRS for back and leg pain and the ODI scores were significantly decreased in both groups. The NRS for back pain and the ODI scores in the PELD + PEEK group (1.32 ± 0.70, 14.10 ± 4.74) were better than those in the PELD group (1.91 ± 0.69, 16.93 ± 4.33) (*P* < 0.05). The IDH of the PELD + PEEK group (10.54 ± 1.62) was significantly higher than that in the PELD group (9.98 ± 1.90) (*P* < 0.05). The IDH of the PELD + PEEK group (10.54 ± 1.62) was significantly higher than that in the PELD group (9.98 ± 1.90) (*P* < 0.05). The IDH of the PELD + PEEK group (10.54 ± 1.62) was significantly higher than that in the PELD group (9.98 ± 1.90) (

**Conclusion:**

For symptomatic patients with GLDH, both PELD and PELD combined with a PEEK rod showed good efficacy. However, the long-term effect of PELD combined with a PEEK rod is better than that of PELD alone. Moreover, PELD combined with a PEEK rod can effectively reduce the recurrence rate. Maximum benefit can be gained if we adhere to strict selection criteria for PELD combined with a PEEK rod.

## 1. Introduction

The incidence of giant lumbar disc herniation (GLDH) accounts for 8–22% of all LDH cases [1–3]. GLDH often causes severe root pain and neurological dysfunction [4]. Percutaneous endoscopic lumbar discectomy (PELD) has been widely used for the treatment of LDH because of its safety and effectiveness, minimal trauma, low interference with the spinal canal, and rapid postoperative recovery [5]. GLDH is a special type of LDH. Extensive intervertebral disc herniation decreases the height of the intervertebral space and causes instability of the spine, which results in chronic lower back pain [6, 7]. Polyetheretherketone (PEEK) rods are widely used as semirigid fixation systems for nonfusion fixation of the spine [8, 9]. These rods can effectively maintain the height of the intervertebral space without the adjacent segment disease (ASD) caused by rigid fixation [10, 11]. Therefore, we used the PEEK rod semirigid fixation system for the treatment of giant disc herniation. This study analyzed the efficacy of a PEEK rod in the treatment of patients with GLDH using a randomized clinical trial that was designed to investigate the efficacy of PEEK rods in alleviating pain and improving function in patients with GLDH.

## 2. Methods

This study is a prospective study and was carried out over 2 years. All the participants provided consent. This study was approved by the Ethics Committee of Chongqing Medical University and registered in the Chinese Clinical Trials Registry (ChiCTR) (Identifier: ChiCTR1900021414).

### 2.1. Patients

**Figure 1:**
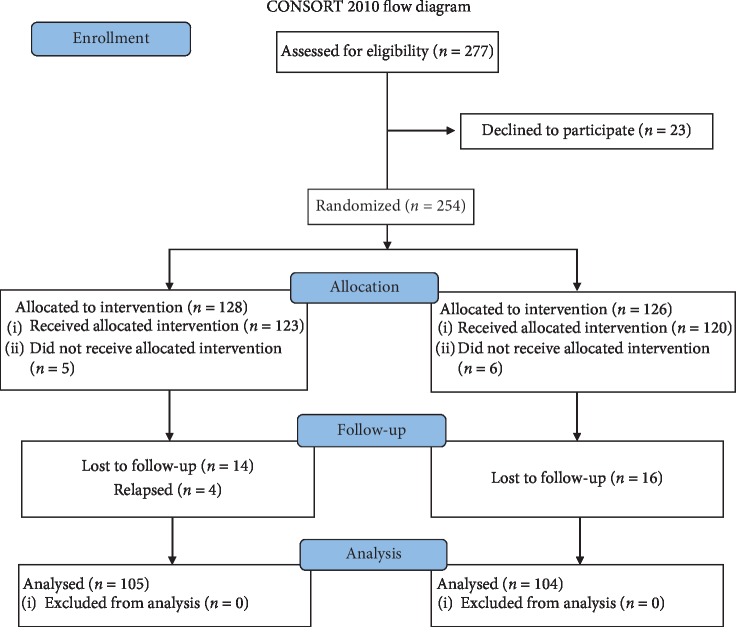
Participant flow diagram.

From June 2015 to June 2017, patients with both back and leg pain were evaluated by magnetic resonance imaging (MRI). Of the 277 patients, 23 patients refused participation in this study, 11 did not receive allocated intervention, and 243 patients were randomly assigned to different groups. Patients were included in the trial after written informed consent was obtained (Figure 1).

The inclusion criteria were as follows: (1) patients with single-level disc herniation and characteristic radiating pain in the leg; (2) preoperative MRI showing massive disc herniation affecting more than 50% of the vertebral canal diameter; and (3) patients who did not respond to more than 6 months of routine conservative treatment, as evidenced by a lack of symptom improvement.

The exclusion criteria were as follows: (1) patients with tumors or fractures in the vertebral body and vertebral body attachments; (2) patients with infections of the skin and soft tissues in the operative field or intervertebral space; (3) patients who previously underwent surgery on the same segments; and (4) patients with severe heart and lung dysfunction who could not tolerate the procedure.

In all, 243 patients were randomly assigned to different groups by generating random numbers with a random number generator so that odd numbers were assigned to the PELD group and even numbers were assigned to the PELD + PEEK rod group. All procedures were completed at the same hospital. The clinical characteristics of the included patients are shown in Table 1.

### 2.2. Surgical Procedure

#### 2.2.1. Group A

Anterior-posterior (AP) fluoroscopy was used to locate and mark the interlaminar space between L5 and S1. A dilator sheath was inserted in the interlaminar space. Then, an operating channel and an endoscope were placed. The endoscope was used to explore the herniated nucleus pulposus tissue and the excision of the herniated disc tissue (if patients did not have L5/S1 disc herniation, we choose the transforaminal approach).

#### 2.2.2. Group B

**Figure 2:**
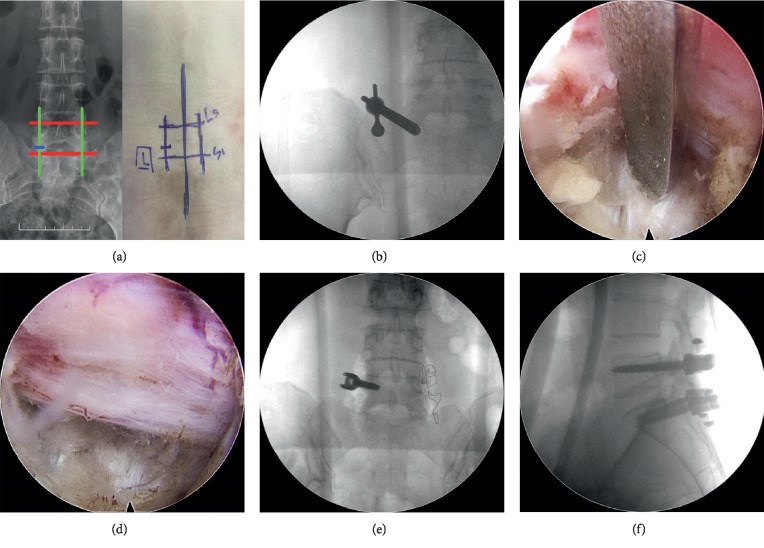
The surgical procedure of PELD combined with a PEEK rod. Red and green lines are the connecting lines of the pedicle, whereas the blue line indicates the location of the interlaminar space (a). An operating channel was placed (b). Exploration of the herniated nucleus pulposus tissue (c). Herniation was removed (d). Pedicle screws were implanted (e). The PEEK rod was placed (f).

First, X-ray fluoroscopy was used to localize and mark the pedicles and the interlaminar space of the target segments (Figure 2(a)). After a longitudinal incision was made, the dilator sheath was inserted into the interlaminar space. Then, an operative trocar and an endoscope were placed (Figure 2(b)). The endoscope was used to explore the herniated nucleus pulposus tissue (Figure 2(c)) and the excision of herniated disc tissue (Figure 2(d)). Two longitudinal incisions were made along the marked lines, exposing the facet via the Wiltse approach (the clearance between the multifidus and longissimus). Then, pedicle screws were implanted (Figure 2(e)) parallel to the endplate. After satisfactory placement of the pedicle screws, the PEEK rod was placed (Figure 2(f)).

### 2.3. Follow-Up

The NRS scores for both back and leg pain and Oswestry Disability Index (ODI) scores were assigned before surgery, and the NRS scores for back and leg pain were reevaluated 1 day after surgery. The NRS and ODI scores were reassessed 3 months, 6 months, 1 year, and 2 years after surgery. All the patients received an X-ray exam of the anterior-posterior and lateral lumbar spine before surgery and 1 day, 3 months, 6 months, 1 year, and 2 years after surgery to assess the IDH. Dynamic X-ray exams were used to evaluate the ROM before surgery and 3 months, 6 months, 1 year, and 2 years after surgery. Lumbar MRI was performed to assess the disc signal in the operated segment and its adjacent segments (if patients had L5/S1 GLDH, we only determined the Pfirrmann grades of the superior adjacent segment) before surgery and 6 months, 1 year, and 2 years after surgery.

The follow-up was terminated if a patient reported recurrent symptoms, and imaging examination indicated reherniation.

### 2.4. Statistical Analysis

Changes in outcome measures from baseline values throughout the entire follow-up period were assessed using a general linear model for repeated measures (SPSS, version 24). A paired *t*-test was used to analyze the NRS and ODI scores in each group before and after surgery. The independent-samples *t*-test was used to analyze the NRS and ODI scores before and after surgery between the different groups. The chi-square test and independent-samples *t*-test were used to compare and analyze the clinical characteristics between the 2 groups. The paired *t*-test was used to analyze the IDH and ROM in each group before and after surgery. The independent-samples *t*-test was used to compare and analyze the IDH and ROM between the 2 groups. The nonparametric Wilcoxon test was used to compare and analyze the within-group and between-group differences in Pfirrmann grades.

## 3. Results

As mentioned above, 277 patients were diagnosed with GLDH. Of these, 23 patients refused participation in this study, 11 did not receive allocated intervention, 30 did not answer phone calls or refused to return to the hospital for follow-up, and 4 patients relapsed (patients reported recurrent symptoms, and imaging examination indicated reherniation). Finally, 209 patients were included in this study.

Patients in both groups successfully underwent surgery. Of those in the PELD group, 4 patients relapsed 2 weeks, 1 month, 4 months, and 5 months after surgery. Per the patients' preferences, 2 patients underwent PELD with dynamic stabilization with a PEEK rod, and 2 patients underwent secondary PELD. In the PELD + PEEK group, no screw loosening or breakage occurred during the follow-up, and no recurrence of disc herniation was reported.

Of the 123 patients in the PELD group, 14 patients were lost to follow-up and 4 patients relapsed. A total of 105 patients completed the follow-up. During the follow-up period, the mean NRS scores for leg and back pain were significantly decreased from 6.99 ± 0.80 and 4.13 ± 0.81, respectively, to 0.84 ± 0.67 and 1.91 ± 0.69 (*P* < 0.001, *P* < 0.001), respectively. The mean ODI score significantly decreased from 75.76 ± 8.11 to 16.93 ± 4.33 (*P* < 0.001). The mean IDH of the surgical segment significantly decreased from 11.33 ± 2.04 to 9.98 ± 1.90 (*P* < 0.001), and the mean ROM of the operated segment significantly increased from 8.21 ± 1.68 to 9.49 ± 1.62 (*P* < 0.001). The Pfirrmann grades were all significantly different before and 2 years after surgery (*P* < 0.05).

Of the 120 patients in the PELD + PEEK group, 16 patients were lost to follow-up. In all, 104 patients completed the follow-up. At the final follow-up, the mean NRS scores for leg and back pain significantly decreased from 6.97 ± 0.79 and 4.13 ± 0.78, respectively, to 0.86 ± 0.67 and 1.32 ± 0.70 (*P* < 0.001, *P* < 0.001), respectively. The mean ODI score significantly decreased from 75.54 ± 8.19 to 14.10 ± 5.88 (*P* < 0.001), and the mean IDH of the operated segment significantly decreased from 11.21 ± 1.91 to 10.54 ± 1.62 (*P* < 0.001). The mean ROM of the operated segment significantly decreased from 8.32 ± 1.76 to 2.39 ± 0.90 (*P* < 0.001). The Pfirrmann grades were all significantly different before and 2 years after surgery (*P* < 0.05). One day after surgery, the mean NRS back pain score significantly increased from 4.13 ± 0.78 to 4.49 ± 0.70 (*P* < 0.001), and the mean IDH of the surgical segment significantly increased from 11.21 ± 1.91 to 11.89 ± 1.68 (*P* < 0.001) (Tables 2–5).

## 4. Discussion

The definition of GLDH varies across the literature and includes a cut-off of greater than 8 mm, herniation affecting 33%, 40%, 50%, or 75% of the sagittal diameter of the spinal canal, or herniation causing complete spinal canal stenosis [1–3, 7, 12–15]. In this study, we defined a herniated disc affecting more than 50% of the sagittal diameter of the spinal canal as giant LDH, which accounts for 8%–22% of all LDH cases [1–3]. Surgical treatment is required when the outcomes of strict conservative treatments are poor or when symptoms are aggravated.

In 2006, Hoogland proposed the transforaminal endoscopic surgical system (TESSYS) technique [16] in which the prominent disc nucleus is directly removed under an endoscope through an intervertebral foramen approach. This approach can reduce damage to the paravertebral muscles, ligaments, and other soft tissues and protect the stability of the spine [17]. In recent years, this technique has been accepted by an increasing number of surgeons.

The PEEK rod-pedicle screw stabilization system, which is a semirigid fixation system, can effectively limit motion of the fixed segments and provide maximal “micromotion” [18–20]. While stabilizing the spine, this system minimizes the risk of ASD caused by rigid fixation.

**Figure 3:**
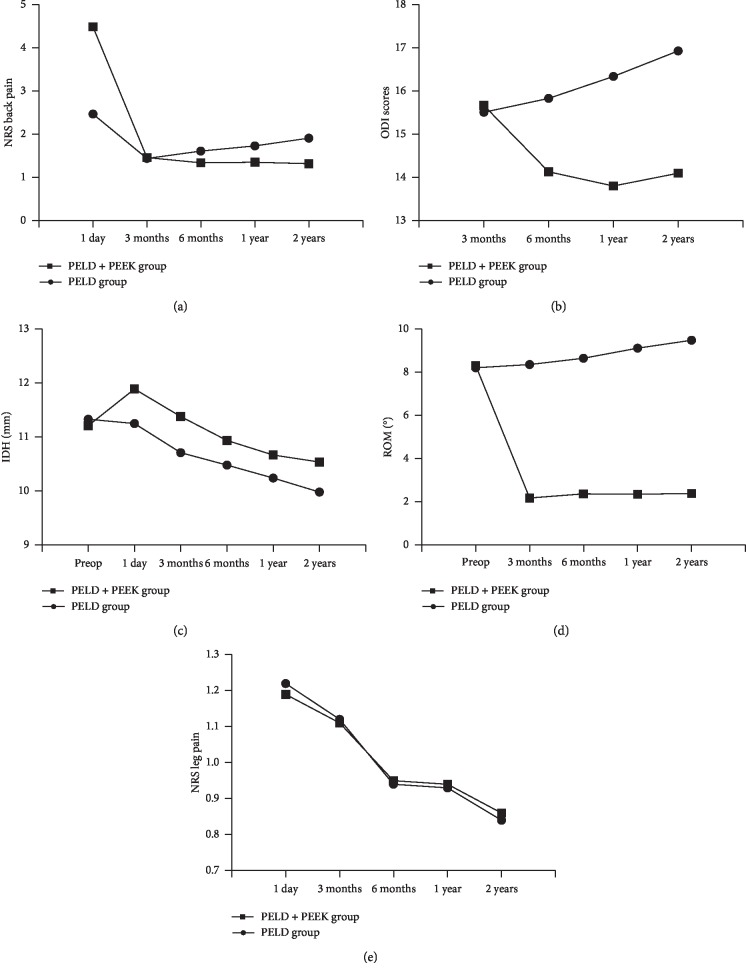
NRS back pain scores (a), ODI scores (b), IDH (c), ROM (d), and NRS leg pain (e). Total score changes for each group during the follow-up.

In this study, the mean NRS scores for back and leg pain as well as the ODI scores in the 2 groups were significantly improved after surgery compared with those before surgery. However, due to the incision in the back, the NRS score of back pain in the PELD + PEEK group was significantly higher than that in the PELD group on day 1 after surgery (*P* < 0.001). The NRS score for back pain and the ODI scores in the PELD + PEEK group were better than those in the PELD group at 6 months, 1 year, and 2 years after surgery (*P* < 0.05) (Figure 3). The authors considered that the above findings may be related to height loss in the intervertebral space and instability of the vertebral body caused by excessive removal of the nucleus pulposus during surgery, which can cause accelerated degeneration of the intervertebral space [8, 9]. Moreover, preoperative X-rays of all patients in this study showed varying degrees of collapse of the intervertebral space between the lesion segments. Excessive lumbar load was transmitted through the facet joints and accelerated degeneration of these joints in the lumbar spine, which resulted in osteoarthritis of the facet joints and joint-derived pain [21]. This is also considered a reason for poor long-term postoperative outcomes.

The IDH of both groups was significantly decreased at the final follow-up. In contrast, in the PELD + PEEK group, the IDH on day 1 after surgery was significantly higher than that before surgery. We believe that the patient position after general anesthesia and the distraction force, when the PEEK rod was placed, can partially distract the vertebral space, and thus, the IDH after surgery was increased temporarily, but then, due to a lack of contents, the IDH gradually decreased. However, at the final follow-up, the IDH of the PELD + PEEK group was significantly higher than that of the PELD group. Therefore, we believe the PEEK rod can help maintain the IDH, and in this way, we can avoid excessive lumbar load transmission through the facet joints. This may be a reason why the NRS back pain scores and the ODI scores in the PELD + PEEK group were better than those in the PELD group.

Ponnappan et al. found that after fixing with a PEEK rod, the mobility decreased from 8.49° to 2.09° in an in vitro test [22]. In the present study, we found that the ROM of the PELD + PEEK group significantly decreased from 8.32 ± 1.76 to 2.39 ± 0.90, which shows that PEEK rods can provide a “micromotion” of the fixed segments in the in vitro test or in vivo in humans. In the PELD group, the ROM significantly increased from 8.21 ± 1.68 to 9.49 ± 1.62. In all, 25 cases of lumbar instability (angle change greater than 11° according to dynamic X-ray) were observed at the final follow-up. This may have caused patients in the PELD group to experience more back pain. Therefore, we suggest that patients with GLDH should undergo fixation with PEEK rods during primary surgery.

In the present study, the Pfirrmann grades in each group were all significantly different between presurgery and the final follow-up (*P* < 0.05). However, the Pfirrmann grades of the surgical segment in the PELD group were significantly higher than those of the PELD + PEEK group at 2 years after surgery (*P*=0.019). This finding suggested that PEEK rods can slow the degeneration of intervertebral discs, possibly because PEEK rods share the load of the intervertebral disc. Additionally, at the final follow-up, the Pfirrmann grades of the adjacent segment of the 2 groups were not different (*P*=0.346; *P*=0.125). Huang W et al. reported that 38 patients who underwent fixation with a PEEK rod did not experience ASD [11] and that patients who underwent fixation with a PEEK rod did not have an increased incidence of ASD. Fay et al. found that younger patients with dynamic fixation had rehydration of the disc after surgery [23]. In the present study, we also found 11 obvious cases of rehydration of the intervertebral disc. Therefore, whether the PEEK rod system is similar to other dynamic fixation systems such as Dynesys in how well it promotes the repair of intervertebral discs is still worthy of further exploration.

In the PELD group, 4 patients relapsed and underwent secondary surgery. The recurrence rate was 3.67%. In the PELD + PEEK group, no patients had relapsed by the end of the follow-up period. Reducing the recurrence rate after PELD remains an issue for spine surgeons. In 2013, Kim et al. performed a follow-up of 18,590 patients with LDH and found that recurrence after PELD usually occurred within 1 year after surgery. If the recurrence rate within 1 year after surgery was reduced, then the overall recurrence rate would be effectively controlled [24]. Therefore, the authors believe that PELD combined with dynamic stabilization with a PEEK rod can effectively reduce the recurrence rate after surgery (*P*=0.049).

Furthermore, we observed an interesting phenomenon. Among the 123 patients in the PELD group, 13 experienced more pain when they stood after surgery. However, in the PELD + PEEK group, 104 patients did not have these symptoms. This phenomenon may also be related to the loss of height of the intervertebral space and instability of the vertebral body caused by excessive removal of the nucleus pulposus.

In the PELD + PEEK group, there is no screw loosening or breakage occurred during the follow-up; however, this randomized controlled study only has a 2-year follow-up, and PELD combined with PEEK robs is a kind of nonfusion fixation surgery; due to fatigue behaviors, the PEEK robs have the risk of breaking. But because of the limitation of follow-up time and patient number, we did not observe any patients with breakage of PEEK robs in our study.

In the present study, we found that PELD combined with dynamic stabilization with a PEEK rod for the treatment of GLDH can achieve better efficacy compared with PELD alone. However, in clinical practice, severe hyperostosis may complicate the intraoperative puncture in many patients. Therefore, to achieve effective decompression, parts of the vertebral body and facet joint bone are removed in many cases, which can interfere with the stability of the spine; therefore, in terms of patient selection, we do not recommend PELD combined with dynamic stabilization with a PEEK rod in older patients or patients with severe hyperostosis/osteoporosis.

## 5. Conclusion

For patients with GLDH, both PELD alone and PELD combined with dynamic stabilization with a PEEK rod can achieve good efficacy. The short-term efficacy of PELD was better than that of PELD combined with dynamic stabilization with a PEEK rod, whereas the long-term efficacy of PELD was inferior to that of PELD combined with a PEEK rod. Furthermore, the recurrence rate in the PELD group was higher than that in the PELD + PEEK group. Patients with GLDH who underwent PELD alone experienced a high probability of future lumbar instability.

## Figures and Tables

**Table 1:** Clinical characteristics of the patients in two groups.

	PELD group	PELD + PEEK group	*P* value
Mean age (years)	45.6 ± 11.6	44.8 ± 11.2	0.573
Sex (male/female)	63/60	61/59	0.952
Level of disc herniation			
L2/3	2	1	0.923
L3/4	3	4	
L4/5	57	55	
L5/S1	61	60	
Preop comparisons			
NRS leg pain	6.99 ± 0.80	6.97 ± 0.79	0.861
NRS back pain	4.13 ± 0.81	4.13 ± 0.78	0.940
ODI scores	75.76 ± 8.11	75.54 ± 8.19	0.824
IDH	11.33 ± 2.04	11.21 ± 1.91	0.646
ROM	8.21 ± 1.68	8.32 ± 1.76	0.670

**Table 2:** Mean preoperative and postoperative NRS (leg and back) and ODI for two groups through the follow-up period.

		Preop	1 day	3 months	6 months	1 year	2 years
NRS leg pain	PELD group	6.99 ± 0.80	1.22 ± 0.78	1.12 ± 0.73	0.94 ± 0.69	0.93 ± 0.68	0.84 ± 0.67
	PELD + PEEK group	6.97 ± 0.79	1.19 ± 0.76	1.11 ± 0.72	0.95 ± 0.69	0.94 ± 0.68	0.86 ± 0.67

NRS back pain	PELD group	4.13 ± 0.81	2.47 ± 0.68	1.44 ± 0.72	1.61 ± 0.70	1.73 ± 0.65	1.91 ± 0.69
	PELD + PEEK group	4.13 ± 0.78	4.49 ± 0.70	1.46 ± 0.77	1.34 ± 0.72	1.35 ± 0.72	1.32 ± 0.70

ODI	PELD group	75.76 ± 8.11	—	15.51 ± 6.20	15.83 ± 4.97	16.34 ± 4.46	16.93 ± 4.33
	PELD + PEEK group	75.54 ± 8.19	—	15.67 ± 6.77	14.13 ± 6.17	13.80 ± 5.46	14.10 ± 4.74

**Table 3:** The radiological outcomes for two groups through the follow-up period.

		Preop	1 day	3 months	6 months	1 year	2 years
IDH (mm)	PELD group	11.33 ± 2.04	11.25 ± 2.03	10.71 ± 2.02	10.48 ± 1.99	10.24 ± 1.96	9.98 ± 1.90
	PELD + PEEK group	11.21 ± 1.91	11.89 ± 1.68	11.38 ± 1.61	10.94 ± 1.62	10.67 ± 1.60	10.54 ± 1.62
ROM (°)	PELD group	8.21 ± 1.68	—	8.36 ± 1.65	8.65 ± 1.57	9.12 ± 1.49	9.49 ± 1.62
	PELD + PEEK group	8.32 ± 1.76	—	2.18 ± 0.97	2.37 ± 1.18	2.35 ± 1.03	2.39 ± 0.90

**Table 4:** Modified Pfirrmann grades of the PELD group and PELD + PEEK group.

Location		Superior segment								Surgical segment								Inferior segment							
Modified Pfirrmann grades		1	2	3	4	5	6	7	8	1	2	3	4	5	6	7	8	1	2	3	4	5	6	7	8
PELD group	Preop	12	42	41	7	3	0	0	0	0	0	5	27	36	33	4	0	4	15	11	4	1	0	0	0
	6 months	11	40	43	8	3	0	0	0	0	0	5	23	35	36	6	0	4	13	12	5	1	0	0	0
	1 year	11	40	42	8	3	1	0	0	0	0	3	24	35	35	5	2	3	12	13	5	2	0	0	0
	2 years	9	38	43	10	4	1	0	0	0	0	1	20	29	40	11	4	3	11	12	6	2	1	0	0
PELD + PEEK group	Preop	12	44	40	6	2	0	0	0	0	0	6	24	39	30	5	0	5	14	12	6	0	0	0	0
	6 months	12	40	41	8	3	0	0	0	0	0	6	24	38	31	5	0	5	13	11	7	1	0	0	0
	1 year	11	38	36	13	5	1	0	0	0	0	5	24	39	30	6	0	3	10	14	8	2	0	0	0
	2 years	10	33	37	15	7	2	0	0	0	0	5	23	37	32	7	0	2	7	14	9	4	1	0	0

**Table 5:** Between-group comparisons made by the independent-samples *t*-test or nonparametric Wilcoxon test (between PELD group and PELD + PEEK group) (*P* value).

	Preop	1 day	3 months	6 months	1 year	2 years
NRS leg pain	0.861	0.803	0.932	0.924	0.924	0.768
NRS back pain	0.940	0.000	0.893	0.006	0.000	0.000
ODI	0.824	—	0.868	0.030	0.000	0.000
IDH	0.646	0.014	0.009	0.073	0.092	0.025
ROM	0.670	—	0.000	0.000	0.000	0.000
Modified Pfirrmann grades						
Superior segment	0.698	—	—	0.832	0.548	0.346
Surgical segment	0.998	—	—	0.474	0.369	0.019
Inferior segment	0.910	—	—	0.934	0.479	0.152

## Data Availability

The data are avalible regarding this study, and can be viewed on ResMan or upon request.
